# Focal Therapy for Localized Prostate Cancer: A Case Series with Cost Analysis

**DOI:** 10.3390/curroncol32090476

**Published:** 2025-08-23

**Authors:** Maxwell Sandberg, David Thole, Jackson Nowatzke, Gavin Underwood, Emily Ye, Soroush Rais-Bahrami, Ronald Davis, Alejandro Rodriguez

**Affiliations:** 1 Department of Urology, Wake Forest University School of Medicine, Winston Salem, NC 27101, USA; david.thole@wfusm.edu (D.T.); jackson.nowatzke@wfusm.edu (J.N.); gavin.underwood@wfusm.edu (G.U.); emily.ye@wfusm.edu (E.Y.); soroush.raisbahrami@advocatehealth.org (S.R.-B.); ronald.l.davis@advocatehealth.edu (R.D.); alejandro.rodriguez@advocatehealth.org (A.R.); 2 Comprehensive Cancer Center, Atrium Health Wake Forest Baptist Medical Center, Winston Salem, NC 27101, USA

**Keywords:** focal, prostate cancer, HIFU, IRE, cryoablation

## Abstract

Focal therapy for prostate cancer (PCa) provides approaches to treat PCa patients in a less invasive manner than traditional whole-gland surgical or radiation modalities. This manuscript provides a case series of high-intensity focused ultrasound (HIFU), cryoablation, and irreversible electroporation (IRE) for PCa at a single institution and cost analysis with a review of the literature. All patients who underwent HIFU, cryoablation, or IRE for localized PCa were retrospectively reviewed, excluding patients who received whole-gland therapy. Functional outcomes were erectile dysfunction and lower urinary tract symptoms. Cost data were collected. A total of 45 patients were included in the study with focal therapy ranging from 2023 to 2025 (4 HIFU, 20 cryoablation, 21 IRE). A total of 30 patients had focally treated lesions, and 15 patients had hemi-gland treatment. The mean preoperative PSA was 7.7 ng/mL. On the paired sample t-test, there was no significant difference between pre-focal and post-focal therapy PSA. Three patients experienced biochemical recurrence requiring prostate biopsy after focal treatment. Mean cost was USD 3804.50 and not significantly different by focal treatment. No metastatic events occurred nor deaths at a median follow-up of 6 months. Patients in this series had largely unaltered functional outcomes. Cost analysis in contemporary publications is lacking. Although follow-up was short, cancer control was adequate.

## Simple Summary

Prostate cancer treatment has shifted dramatically over the last decade. In this manuscript, a case series of three different focal therapy modalities (high-intensity focused ultrasound, cryoablation, and irreversible electroporation of the prostate) to treat prostate cancer is reported. The analysis compares functional and oncologic controls. Focal therapy provides adequate functional outcomes with oncologic control like established whole-gland therapies. Additionally, the cost by treatment modality is compared, often disregarded when considering focal therapy. Lastly, a brief narrative review of the literature was conducted to contextualize our findings.

## 1. Introduction

The annual rate of incidence of prostate cancer (PCa) in the United States of America (USA) was over 299,000 in the year 2024 [1]. PCa treatments have been revolutionized over the last decade, with improved survival rates. In 2024, the five-year cancer-specific survival (CSS) was estimated to be 97% in the USA alone [1]. Traditional treatments for PCa have been radical prostatectomy (RP) and radiation to the prostate, each of which is a whole-gland therapy with curative intent, still widely employed in practice today. The literature varies, but approximately 40–60% of men opt for RP as the primary means to treat their PCa [2,3,4]. The average number of RP performed in the USA has remained relatively steady but difficult to estimate, at around 138,000 RP performed each year [5]. However, only a minority of urologists are high-volume surgeons performing the majority of RP [6]. The second most common treatment selection is radiation therapy, either external beam radiation or brachytherapy approaches directed by radiation oncologists, with approximately 37% of men with PCa receiving primary radiation therapy each year for their disease [4]. Both RP and radiation therapy carry significant side effects to patients. These are most often urinary incontinence and erectile dysfunction (ED). ED and urinary incontinence can impact upwards of 30% and upwards of 70–80% of patients after RP, respectively [7]. Further, evidence has pointed to around 30% of patients experiencing urinary incontinence and 30–50% of patients experiencing ED after radiation therapy [8]. Only ~5–10% of PCa patients elect active surveillance in the USA, although the number is significantly higher for low-risk disease, with some estimating 60% [9,10]. This is particularly relevant as 10–30% of patients with localized PCa report regret in the way they managed their PCa [11,12]. There are multiple reasons for this but given there is still a 2.6% risk of dying of PCa, many men want to take a more active role in treating their disease but may not be aware of the side effects with the standard whole-gland treatment options [13]. Additionally, the increased use of prostate indication magnetic resonance imaging (MRI) and MRI-guided biopsy through software fusion technologies has augmented the reliability of grade and stage-defining diagnostic biopsy samples, with more confident decision making when suitable for surveillance versus definitive therapy of varying levels of aggressiveness [14,15,16].

Focal therapy with various ablative energy sources has created a revolutionary approach to managing PCa in a less invasive way. Subtotal gland treatments can use a variety of different energy modalities, all with the fundamental goals of treating index lesions of known, localized PCa while sparing unaffected tissue regions in the gland [17]. Often, this includes but is not limited to high-intensity focused ultrasound (HIFU), cryotherapy, and irreversible electroporation (IRE) of the prostate which have long been used and vetted for whole-gland prostate ablative approaches [18]. There is a paucity of research comparing these different modalities, and no randomized clinical trial exists comparing them head-to-head with standard-of-care, whole-gland curative treatment options (RP or radiation therapy) [19]. Current case series seem to show promising results for HIFU, cryoablation, and IRE with respect to functional outcome preservation in focal treatments [19,20,21]. Cancer control evidence is not as strong for focal treatments, but case series in the literature seem to show no difference in overall survival (OS) and metastasis-free survival (MFS) compared to RP and radiation [19]. Nevertheless, additional patient data is required, and cost is not factored into many of the contemporary publications of these treatment modalities. The purpose of this paper is to report a case series of focal/hemi-gland HIFU, cryoablation, and IRE with respect to functional outcomes and cost at a single institution and provide a brief narrative review of the literature surrounding these treatment modalities. Secondarily, the purpose was to report short-term oncologic outcomes.

## 2. Materials and Methods

This was a retrospective study from 2023 to 2025 performed at a single institution of all patients who underwent either HIFU, cryoablation, and/or IRE for localized PCa. All patients who received whole-gland therapy were excluded, such that only focal and/or hemi-gland therapy was included in the analysis. The study was approved under the Wake Forest Baptist Institutional Review Board (IRB00132775). Demographic information was collected on all eligible participants including age, race, comorbidities, and Charlson Comorbidity Index (CCI) at the time of focal therapy administration. Functional status at baseline was tracked for both ED and lower urinary tract symptoms (LUTS). This was achieved in the form of both qualitative documentation of ED and LUTS preoperatively as well as preoperative International Index of Erectile Function (IIEF) scores and International Prostatic Symptom Scores (IPSS). Additionally, complications were recorded after focal therapy and graded using the Clavien-Dindo classification system [22]. Preoperative oncologic data were also collected which included prior prostate biopsy data with grade group (GG) prior to therapy, prostate-specific antigen (PSA) value prior to focal treatment, prostate size on MRI, and prior history of focal treatment and/or other treatment for PCa. Patients were also categorized based on PCa risk category using the American Urologic Association Classification System [23]. Patient selection for focal therapy was at surgeon discretion and not standardized in the study. However, it was most often based on GG and PCa risk stratification, with a specific focus on GG 1–2 and risk category ≤ favorable intermediate risk. All patients were required to have a pre-focal therapy MRI completed, most often within 3 months or less of focal therapy administration, all of which were multiparametric MRIs. There were no absolute exclusions by tumor location, but multiple positive locales on preoperative prostate biopsy were typically justified by a surgeon for hemi-gland over focal gland treatment. There were no criteria to choose one energy modality over another (i.e., cryoablation versus IRE, etc.). However, HIFU was only recently introduced to this institution in 2024, which accounts for its making up the smallest proportion of patients in the study. HIFU was performed using Sonablate^®^ (Charlotte, NC, USA), IRE was performed using NanoKnife systems, and cryoablation was performed through Endocare™ (CryoCare CS^®^, Winston Salem, NC, USA) and Galil (Boston Scientific, Winston Salem, NC, USA) probes.

Postoperative oncologic data recorded included operative time, most recent follow-up PSA, need for additional prostate biopsy and biopsy data, development of metastasis, most recent follow-up, and OS. All patients were seen in the clinic postoperatively 4–6 weeks after focal therapy administration with a PSA lab draw. Then, a PSA was followed at 3, 6, and 12 months post-procedure. An MRI was performed one year post-therapy. A post-treatment confirmatory biopsy was performed based upon the PSA and MRI results, specifically using MRI targeting of any sites of suspicion and the site of post-ablative tissue involution changes. PSA was then checked every 6 months up to 5 years post-focal therapy. The postoperative PSA median follow-up interval was six months. Cost data was obtained from medical record logs of each case and comprised healthcare system costs, not costs to the patient. This included operating room disposable costs such as supplies, machinery (which was rented now owned), and the like. Operating room block times and anesthesia costs were not included. Not every case had available cost data, so only cases with cost information (IRE and cryoablation) were included in the analysis on cost. Additionally, not every patient had a complete set of data available in the electronic medical record. Independent sample t-test was utilized to compare the mean cost between IRE and cryoablation. Analysis of variance was used to compare operative time by treatment modality. Paired sample t-test was used to compare pre-focal to post-focal therapy PSA values both in the overall cohort and for each individual treatment type. All statistical analysis was performed using SPSS Statistics Version 28 (Armonk, NY, USA).

In addition to reporting a case series, this study performed a brief review of the literature and summarized these findings in the discussion of the paper. Further, the data from this case series were compared in descriptive format to some current publications.

## 3. Results

**Figure 1:**
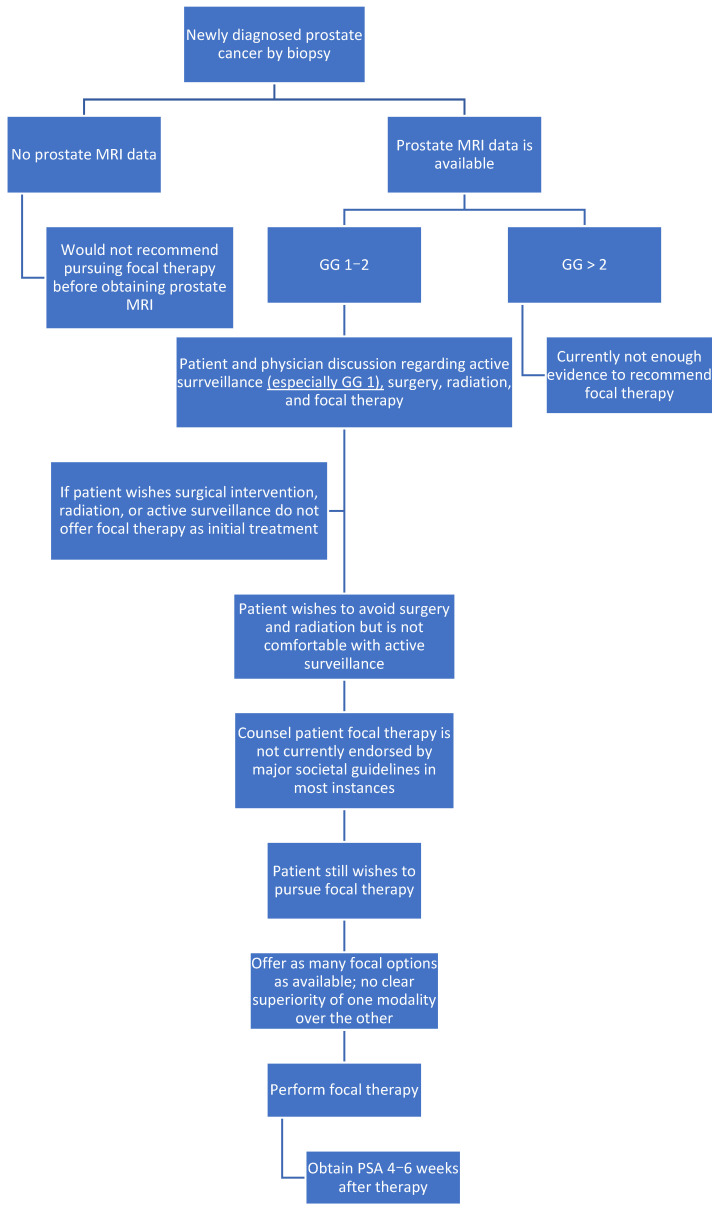
Focal therapy algorithm. The following image displays an algorithm generated by the authors of this manuscript to guide urologists considering focal therapy for a patient with prostate cancer. It is not meant to represent level I evidence nor expert-agreed fact. This algorithm also does not address salvage focal therapy in the setting of previously treated prostate cancer.

A total of 45 patients were included in the analysis (4 HIFU, 20 cryoablation, and 21 IRE; Table 1). Of these, 30 patients had focally treated lesions, and 15 patients had hemi-gland treatment. Median follow-up was 6 months in the study. The mean age at the time of focal therapy was 70.1 years old. There were 34 (76%) Caucasian patients, 8 (18%) patients who were black, and 3 (7%) patients whose race was listed as “other.” Mean CCI was 5.7 at the time of focal therapy. A total of 13 (29%) patients received prior treatment for their PCa. Of these, two had brachytherapy, four had radiation therapy, six had prior cryoablation, and one had prior IRE. Focal therapy is not infrequently administered in the salvage setting after prior treatment for PCa rather than as a primary treatment, with promising outcomes for use in this setting; thus, it was deemed justified to include these patients in the study [24,25,26]. Further, the goal of this paper was to report a case series of HIFU, cryoablation, and IRE which provided additional justification for their inclusion. There were 27 (60%) patients with ED preoperatively and 16 (36%) patients noted to have ED postoperatively. The mean preoperative IIEF score was 14.7, and the mean postoperative IIEF score was 7. Two patients did not have ED pre-focal therapy and were found to have developed ED post-focal therapy in the study (1 IRE and 1 cryoablation). There were 23 (51%) patients noted to have LUTS preoperatively and 20 (44%) patients with LUTS postoperatively. The mean preoperative IPSS was 7.9, and the mean postoperative IPSS was 6. Five patients with no LUTS pre-focal therapy developed LUTS post-focal therapy (two IRE and three cryoablation). The mean prostate size at the time of treatment was 46.1 cc. Figure 1 displays an algorithm for urologists to use when considering focal therapy for a patient with PCa.

Mean preoperative PSA was 7.7 ng/mL. There were 5 (11%) patients with GG 1 PCa preoperatively, 30 (67%) patients with GG2 PCa, 9 (20%) patients with GG3 PCa, and 1 (2%) patient with GG4 PCa. There were 4 (9%) patients with very low risk PCa, 35 (78%) patients with favorable intermediate risk PCa, 9 (20%) patients with unfavorable intermediate risk PCa, and 1 (2%) patient with high risk PCa preoperatively in the cohort. The mean operative time was 59.2 min overall, with a significant difference by focal modality with HIFU at 114.3 min, cryoablation at 65.2 min, and IRE at 43.1 min (*p* < 0.05). Overall, there were 16 (36%) complications reported postoperatively. Of these, only two were > Clavien-Dindo II, and both were IIIa. The most recent mean PSA on follow-up was 3.1 ng/mL. On the paired sample t-test, there was no significant difference between pre-focal and post-focal therapy PSA (*p* > 0.05; Table 2). In individual focal therapy analysis, both IRE and cryoablation had a significantly lower PSA post-focal therapy compared to pre-focal therapy (*p* < 0.05), but HIFU did not (*p* > 0.05). There were three patients who underwent postoperative biopsy after focal therapy during the study window, all of whom were cryoablation patients, and all of whom were positive for PCa. All biopsies (100%) were GG2 disease. No patients died during the study window nor did anybody develop metastasis.

Cost data was only available for IRE and cryoablation patients. Mean cost of IRE was USD 3762.30 and mean cost of cryoablation was USD 4648.30 (*p* > 0.05).

## 4. Discussion

Currently, focal therapy is not endorsed by the American Urologic Association and National Comprehensive Cancer Network (NCCN) guidelines to treat PCa for any risk level, aside from select instances of radio-recurrent PCa [23,27,28]. If a patient fails radiation therapy for PCa and has a recurrence, whole-gland cryoablation and HIFU are considered for salvage treatment in the NCCN guidelines [27,28]. The European Association of Urology guidelines note that HIFU and cryoablation may be considered for select patients as part of a clinical trial or prospective cohort study [29,30]. Recently, the FocAL therapy CONsensus (FALCON) project released some recommendations to refine partial gland ablation for localized prostate cancer [31,32]. Although no consensus or significant societal endorsement exists for focal therapy in PCa guidelines, many urologists employ these treatment modalities in select cases, largely with the driver of patient desire and the advances in pretreatment imaging defining the isolated localization of clinically significant PCa foci [33,34].

There is a paucity of data on the exact percentage of patients opting for focal therapy to treat their PCa, with some critics noting its use in improperly selected patients. In Germany, Flegar et al. analyzed the German Billing Database for focal therapy over the course of 2006–2019 for hyperthermia ablation, cryoablation, vascular-targeted photodynamic therapy, transurethral ultrasound ablation, and various other modalities [35]. They found nearly 24,000 focal therapy cases for PCa performed in this time frame, with an initial increase and then a plateau in use around the year 2015. However, for patients >70 years old undergoing focal treatment for PCa, they noted a significant increase over time in its use [35]. Interestingly, the mean age in this series was >70 years old. Multiple critics of focal therapy argue that it is too often employed to treat disease in the wrong patients or undertreats clinically significant PCa [36,37]. Tay et al. performed a systematic analysis of HIFU, cryoablation, and IRE [38]. They noted an increasing trend of these treatments being used for GG2-5 disease and a downtrend in their use for GG1 PCa in their study. At this institution, there has been an uptick in HIFU, which was only added as an option for patients under care early in 2024, as well as increased utilization of IRE. Further, only 11% of this case series received treatment for primary pattern GG1 disease. The data highlights the need for large population-based studies to identify how frequently focal therapy is being employed and in which patients urologists are opting to use it on.

Functional outcomes were adequate, like much of the current literature. Proponents of treatments like HIFU, cryoablation, and IRE argue improved ED and lesser degrees of LUTS relative to radical surgery or radiation in PCa [37]. Just two patients who did not have ED preoperatively were found to have developed it after therapy. Tay et al. performed a systematic review of 49 HIFU, cryotherapy, and IRE studies, of which 35 reported sexual function data [38]. The overwhelming majority of studies reported a low to moderate impact of all three treatments on sexual function, with only two studies reporting severe sexual side effects. Additionally, no significant differences were identified between each of the three treatments. While this study did not directly compare each focal modality in the case series, similar proportions of patients with ED pre- and post-focal therapy by treatment modality were identified. Another review by Hopstaken et al. included 27 HIFU studies, 11 on cryoablation, and 9 on IRE [39]. These authors broke HIFU down to a prior history of transurethral resection of the prostate (TURP) versus no prior TURP, as some perform TURP initially to reduce the risk of urinary retention. For HIFU with TURP, sexual function was mixed, with some authors noting a moderate increase in ED after HIFU, while others showed a return to baseline one year postoperatively [40,41,42]. For patients who had HIFU and no prior TURP history, a median decrease in erectile function of 12% was noted after HIFU, and there was a median increase in the use of phosphodiesterase five inhibitors (17%) after treatment [39]. No patients in this HIFU series had a TURP prior. For IRE, six of the nine studies in Hopstaken’s review noted ED after therapy, and 50% of these reported severe dysfunction [39]. One important analysis by Mendez et al. compared focal to whole-gland cryoablation, noting significantly improved sexual function in focally treated patients, with nearly 50% reporting early return to sexual activity [43]. Tan et al. published on 28 patients who underwent focal cryoablation, concluding there was a transient decline in sexual function postoperatively, but this recovered within three months [44]. Rastinehad et al. found that out of 282 focal cryoablation patients, 74% of patients potent prior to therapy maintained erections necessary for sexual intercourse after cryoablation [45]. In this series, 67% of patients received focal gland therapy compared to 33% hemi-gland.

Urinary outcomes for focal HIFU, cryoablation, and IRE vary across the literature, but appear comparable to those of RP or radiation. In this study’s cohort, nearly identical rates of LUTS before and after focal therapy were seen. Additionally, only two patients who did not report LUTS before focal therapy were found to have developed LUTS postoperatively. In ten studies of TURP followed by HIFU, 95% of patients were pad-free after HIFU [39]. For HIFU without TURP, nearly 96% of patients in six different studies were pad-free after treatment [39]. IRE had even better outcomes in the analysis by Hopstaken et al., with a 100% pad-free rate. Other contemporary series on IRE report continence rates also approaching 100%, although one small series of elderly patients noted pads to be required in 18% of patients more than six months postoperatively [46]. Looking at cryoablation, Hopstaken et al.’s meta-analysis had five focal studies reporting urinary continence outcomes, of which four noted 100% pad-free continence after therapy [39]. Mendez et al. compared focal to whole-gland cryoablation, finding improved urinary continence rates in the focal grouping [43]. Rastinehead et al. recently presented a cohort of 282 patients who underwent focal cryoablation for PCa, with a 98% pad-free rate at 12 months postoperatively [45]. In summary, it appears IRE offers the highest rates of continence postoperatively of the three modalities, but all provide adequate outcomes in this domain. IRE patients in this series had outcomes in line with this, but given the small cohort and follow-up, no definitive comments on the superiority of one focal therapy over another using these data can be made.

Oncologic outcomes for HIFU, cryotherapy, and IRE are difficult to assess due to significant heterogeneity in the current study design and patient selection. Nevertheless, the current landscape seems to indicate that outcomes are adequate. In Tay et al.’s systematic analysis of HIFU, cryoablation, and IRE, 22 different studies reported survival outcomes, with 98% OS noted [38]. CSS was 99.3%. MFS was 98.5%, and no difference in OS, CSS, or MFS was observed by focal modality [38]. Additionally, biochemical recurrence was noted to be approximately 9% per year. Ślusarczyk et al. performed a meta-analysis of prospective focal therapy studies, which included 50 studies, of which 18 were HIFU, 11 cryoablation, and 7 IRE [47]. The 12-month biopsy-proven recurrence-free survival was 81% and was not different by focal therapy type. MFS differed based on median follow-up in the study but ranged from 93 to 100%. Most research did not directly compare oncologic outcomes by focal therapy type, but Stabile et al. compared HIFU to cryoablation and did not identify oncologic superiority of one modality over the other [48]. No deaths occurred in this analysis’s study window nor metastatic events. The short follow-up time and heterogeneous cohort make oncologic analysis complicated. The overall body of literature, however, appears promising.

Adverse events in Ślusarczyk et al.’s study found complications ≥ Clavien III in approximately 3% of instances, with no difference by focal modality [47]. Hopstaken et al. reported a median ≥ Clavien III complication rate of 1.9% in HIFU with TURP, 2% in HIFU without TURP, cryoablation ranged with most studies noting 0–1.6%, and nearly 0% for IRE [39]. In this study, a complication rate of 35% was noted, but only 4% of complications were ≥Clavien III, all of which were IIIa. Given that RP has a complication rate ≥ Clavien III around 9%, focal therapy appears to be a safe alternative for clinicians to employ [49].

One of the main issues with focal ablative therapy is the lack of level one evidence to allow for adoption in the large panel-derived guideline bodies that define its role in the treatment of PCa. No clinical trial has ever been published directly comparing focal therapy to RP or whole-gland radiation therapy. The only randomized clinical trial to compare focal therapy to standard-of-care treatments in the space of PCa evaluated 206 men who received vascular targeted photodynamic therapy of the prostate to 207 men on active surveillance [50]. There was a significantly lower rate of disease progression in the phototherapy group relative to active surveillance and at a four-year lower rate of requiring radical therapy [50,51]. Clinical trial design is difficult. The correct target population to include in the ideal clinical trial remains elusive. Additionally, as noted by others, even the best focal therapy studies have only modest population sizes [52]. Further, treating localized tumor lesions does not necessarily lead to meaningful survival outcomes [52]. Questions also remain regarding future urologists using focal therapy in their practices, as little data exists on how often urologic residents receive exposure and if they are even trained in it at all.

A unique aspect of this case series is the inclusion of cost data. Most of the cost information came from IRE, which was found not to significantly differ from cryoablation. Consideration should be given to the difference in operative time, appreciated though, as IRE had the shortest operative time. Reddy et al. utilized a Markov Model to model stable disease, local recurrence, metastatic disease, and death from PCa [53]. Focal therapy was defined as either cryoablation or HIFU, and radical therapy was RP or radiation. Interestingly, focal therapy had lower overall costs and higher quality-adjusted life year gains compared to RP or radiation [53]. Silva and Lima calculated that it costs around USD 1100 to treat a man with PCa using HIFU [54]. There is surprisingly little published data comparing focal therapy modalities by cost to one another. Given that there does not appear to be a meaningful difference by focal therapy regarding functional outcomes nor cancer control, cost should be a high priority when considering treating a patient with either HIFU, cryoablation, or IRE.

Multiple limitations exist in this series worth acknowledging. First, while the majority of the literature is mainly small cohorts of focal therapy patients, this study also has relatively small numbers of patients who received HIFU, cryoablation, and IRE. Moreover, the short interval of follow-up significantly limits the study’s ability to analyze functional and oncologic outcomes. The limited follow-up particularly limits oncologic data, as it is unexpected that relevant outcomes like recurrence or metastasis would frequently happen in the 6-month median follow-up time. Focal therapy is relatively new and has been slow to be adopted in mainstream practice, and short follow-up times are relatively ubiquitous in the literature, with many studies reporting median follow-ups of less than two years [24]. An updated series with lengthier follow-ups would be useful in the future to report on. A complete set of data, both pre- and post-focal therapy on each patient (i.e., IIEF scores, IPSS, etc.) for the entire series, was not available, given the retrospective nature of the data collection. Additionally, there are other ablative modalities used for focal therapy that are not included in the current analysis and literature review since they are not currently offered in this institution’s practice including laser ablation, focal brachytherapy, focal stereotactic body radiation therapy, and partial prostatectomy. The cost information included is a strength of this series, but no cost data were available for HIFU patients, and minimal data were available on cryoablation. Further, costs were mainly representative of operating room disposables and machinery rentals, so it is likely the analysis underestimates the true total cost of focal therapy. Moreover, given that wage levels, material prices, and other factors vary across countries and regions, the conclusions of this study only represent the results from a single institution where the study was conducted. Therefore, the cost significance of this study is limited. Still, given the paucity of cost analysis in the focal therapy literature, there is still value in this data which can guide more robust studies in the future.

## 5. Conclusions

In this case series, outcomes of treating localized PCa patients with either HIFU, cryoablation, or IRE at a single institution were presented. Additionally, a comprehensive synopsis of the current literature surrounding these focal treatment options was conducted. Outcomes show focal therapy to be feasible, with excellent functional outcomes and adequate oncologic control. Moreover, cost was analyzed. The literature shows similar success with HIFU, cryoablation, and IRE. What is clear from this series and review is that more level one evidence evaluating focal therapy compared to the standard of care in PCa is required before fully implementing these therapies into guidelines. Nevertheless, they represent an exciting new avenue for future research, and focal therapy seems poised to change the landscape of PCa management moving forward.

## Figures and Tables

**Table 1:** Focal therapy series data. The following table shows different patient and oncologic variables in the left-most column. Each focal modality is shown with corresponding values for each variable. Continuous variables are means with standard deviations in parentheses. Categorical variables are total numbers with percentage of the cohort in parentheses. Total represents the entire focal therapy cohort.

Variable	HIFU	IRE	Cryoablation	Total
N	4	21	20	45
Age	66.5 (13.3)	70.5 (7.4)	70.6 (7.4)	70.1 (8.2)
Caucasian	3 (75)	16 (76)	15 (75)	34 (76)
Black	1 (25)	5 (24)	2 (10)	8 (18)
Other	0	0	3 (15)	3 (7)
Charlson Comorbidity	4 (5.0)	5.7(1.7)	5.9 (2.0)	5.7 (1.9)
IIEF preoperative	19.5 (0.7)	13.6 (7.3)	14.7 (8.3)	14.7 (7.4)
ED preoperative	1 (25)	12 (57)	14 (70)	27 (60)
IPSS preoperative	7.3 (4.6)	8.1 (3.5)	8.0 (7.4)	7.9 (5.2)
LUTS preoperative	2 (50)	11 (52)	10 (50)	23 (51)
Focal	1 (25)	17 (81)	12 (60)	30 (67)
Hemi	3 (75)	4 (19)	8 (40)	15 (33)
Prior treatment				
Brachytherapy	0 (0)	0 (0)	2 (10)	2 (15)
Radiation	0 (0)	1 (5)	3 (15)	4 (31)
Cryoablation	0 (0)	2 (10)	4 (20)	6 (46)
IRE	0 (0)	0 (0)	1 (5)	1 (8)
Preoperative PSA (ng/mL)	7.2 (2.2)	7.0 (3.0)	8.5 (3.8)	7.7 (3.3)
GG preoperative				
1	0 (0)	2 (10)	3 (15)	5 (11)
2	3 (75)	13 (62)	14 (70)	30 (67)
3	1 (25)	5 (24)	3 (15)	9 (20)
4	0 (0)	1 (5)	0 (0)	1 (2)
Prostate size (cc)	52.8 (17.3)	51.3 (25.4)	39.5 (17.0)	46.1 (21.7)
Very low risk	0	2	2	4 (9)
Favorable intermediate risk	5	13	17	35 (78)
Unfavorable intermediate risk	1	5	3	9 (20)
High risk	0	1	0	1 (2)
Operative time (minutes)	114.3 (20.6)	43.1 (6.1)	65.2 (14.0)	59.2 (23.3)
Complication	1 (25)	7 (33)	8 (40)	16 (36)
Clavien				9 (20) 6 (13) 2 (4)
I	0 (0)	4 (19)	5 (25)	
II	2 (50)	3 (14)	1 (5)	
IIIa	0 (0)	0 (0)	2 (10)	
IIIb	0 (0)	0 (0)	0 (0)	
IV	0 (0)	0 (0)	0 (0)	
V	0 (0)	0 (0)	0 (0)	
Most recent PSA (ng/mL)	4.5 (2.4)	3.5 (2.4)	2.5 (2.2)	3.1 (2.3)
Biopsy postoperative	0 (0)	0 (0)	3 (15)	3 (7)
Positive biopsy postoperative	0 (0)	0 (0)	3 (15)	3 (7)
GG postoperative	-	-	3 (100)	3 (100)
IIEF postoperative	-	14 (67)	7	10.5 (5)
ED postoperative	0 (0)	4 (19)	12 (60)	16 (36)
IPSS postoperative	5	0	6.3 (6.8)	6 (5.6)
LUTS postoperative	0 (0)	7 (33)	13 (65)	20 (44)
Cost (USD)	-	3762.3 (2552.3)	4648.3	3804.5 (2495.2)
Metastasis	0	0	0	-
Dead	0	0	0	-
Follow-up (months)	4 (3–8)	4 (4–7)	12 (2.5–17)	6 (3–12)

**Table 2:** Paired samples t-test for PSA. The following table compares pre-focal therapy PSA to post-focal therapy PSA values. Each individual treatment modality is shown along with the total cohort. *p*-values are provided for reference.

Therapy	PSA Preoperative (ng/mL)	PSA Postoperative (ng/mL)	*p*-Value
HIFU	6.4 (1.9)	4.5 (2.4)	0.082
IRE	7.1 (3.3)	3.5 (2.4)	0.001
Cryoablation	8.0 (3.8)	2.5 (2.2)	<0.001
Total	7.5 (3.4)	3.1 (2.3)	0.854

## Data Availability

Data is not publicly available due to patient privacy, but will be made available in a de-identified format upon reasonable request to the corresponding author.

## Abbreviations

The following abbreviations are used in this manuscript:

PCa	Prostate cancer
USA	United States of America
RP	Radical prostatectomy
ED	Erectile dysfunction
MRI	Magnetic resonance imaging
HIFU	High-intensity focused ultrasound
IRE	Irreversible electroporation
OS	Overall survival
MFS	Metastasis-free survival
LUTS	Lower urinary tract symptoms
IIEF	International Index of Erectile Function
IPSS	International Prostatic Symptom Score
GG	Grade group
NCCN	National Comprehensive Cancer Network
FALCOS	FocAL therapy CONsensus
TURP	Transurethral resection of prostate
CSS	Cancer-specific survival
